# The Biological Response of Human NP Cells to Soluble Collagen VI

**DOI:** 10.3390/cells15151339

**Published:** 2026-07-26

**Authors:** Jonathan Dalton, Srikanth N. Divi, Yasmine K. Eichbaum, Panya Luksanapruksa, Mitchell K. Ng, Rachel Huang, Alan S. Hilibrand, Alexander R. Vaccaro, Dessislava Z. Markova, Christopher K. Kepler

**Affiliations:** 1 Department of Orthopaedic Surgery, Thomas Jefferson University, Philadelphia, PA 19107, USA; 2 Department of Orthopaedic Surgery, Rothman Institute, Philadelphia, PA 19107, USA; 3 Department of Orthopedic Surgery, Northwestern Hospital, Chicago, IL 60611, USA; 4 Department of Orthopedic Surgery, Siriraj Hospital, Mahidol University, Bangkok 10700, Thailand

**Keywords:** intervertebral disc degeneration, collagen type VI, nucleus pulposus, degenerative disc disease

## Abstract

**Highlights:**

**What are the main findings?**

- ColVI expression increases with higher Pfirrmann grades.
- Soluble ColVI stimulates nucleus pulposus (NP) cell proliferation and increases IGF-1 expression; this IGF-1 expression was reduced with ERK inhibition.

**What are the implications of the main findings?**

- This mitogenic signaling, induced by ColVI, may contribute to the development of intervertebral disc disease.
- Further elucidation of these pathways may provide therapeutic targets to slow or prevent IVD degeneration.

**Abstract:**

Extracellular matrix (ECM) dysregulation plays a significant role in the development of intervertebral disc disease (IVDD). The ECM around the chondrocytes in the nucleus pulposus (NP) is unique in that it contains ColVI. While linked to chondrocyte proliferation in cartilage, ColVI’s specific role in IVD degeneration is less clear. Our aim was to determine the expression of ColVI in NP cells from degenerative IVDs, along with the effects of soluble ColVI on NP cells. Thus, we compared the expression levels of ColVI in human degenerated IVD samples, along with the effect of soluble ColVI on primary human NP cells. A higher Pfirrmann grade was associated with increased *COL6A1* mRNA expression, with a 4.4-fold increase in Pfirrmann grade IV (*p* = 0.024) and a 5.3-fold increase in grade V compared to grade III (*p* = 0.014). Soluble ColVI increased NP cell proliferation compared to untreated controls, from 1.204-fold for 5 µg/mL (*p* < 0.001) and 1.335-fold for 10 µg/mL (*p* = 0.001). The most upregulated gene was IGF-1 (7.9-fold increase). Co-treatment of NP cells with ColVI and an ERK inhibitor showed significantly less *IGF-1* mRNA upregulation compared to ColVI treatment alone (6.2-fold vs. 12.4-fold; *p* = 0.009). Our findings suggest that degenerating discs are associated with COL6A1 upregulation by NP cells; moreover, the addition of soluble ColVI to NP cells increases NP cell proliferation and IGF-1 expression.

## 1. Introduction

Intervertebral disc degeneration (IVDD) is a prevalent cause of discogenic low back pain (LBP) and disability in the aging population, and includes stenosis, degenerative spondylolisthesis, and scoliosis [1,2,3]. LBP is estimated to cost over $100 billion annually in the United States via direct/indirect expenditures [4]. While studies have traditionally focused on environmental exposures such as smoking and heavy labor, newer research has emphasized the role of genetic factors in the development of IVDD [5]. Despite this, the definitive molecular mechanisms of IVDD remain unclear.

The IVD is composed of a tough peripheral annulus fibrosus (AF) that can withstand tensile strain, and a gelatinous central nucleus pulposus (NP) that resists compression [5]. Both regions have a relatively small population of chondrocytes and an abundance of extracellular matrix (ECM) components, including collagen, aggrecan, and other small proteins [5,6,7]. The ECM immediately surrounding chondrocytes in the AF and NP, the pericellular matrix (PCM), regulates replication, mobility, and adhesion [8,9,10]. The PCM is uniquely enriched in collagen Type VI (ColVI), and within articular cartilage, the PCM and chondrocyte unit form chondrons [7,11,12]. In the IVD, chondrocyte-like cells are surrounded by the PCM with substantial ColVI content [13]. ColVI has been shown to interact with collagen Type II, integrins, aggrecan, and other receptor proteins to anchor chondrocytes within the larger ECM and to perform mechano-transduction [14,15,16]. Early work showed that ColVI decreased in IVDD and adopted a more diffuse rather than pericellular pattern [11]. *COL6A2*, one of the three subunits of the trimeric ColVI molecule, has also been shown to be important for IVD cell survival and proliferation via the PI3K-Akt signaling pathway [17]. *COL6A2* mRNA is significantly greater in degenerative samples of IVD and has been identified as a key gene associated with the pathogenesis of IVDD [17]. However, the degree to which *COL6A2* in particular, and ColVI in general, are either markers or drivers of the NP degeneration cascade is not conclusively known [17,18,19,20,21,22]. The application of soluble ColVI has been shown to increase chondrocyte proliferation in adult and osteoarthritic tissue, cardiac myofibroblast differentiation, and hepatic stellate cell proliferation [23,24,25]. These same findings are not seen with the application of immobilized ColVI, indicating that soluble ColVI may be a therapeutic target in degenerative disease [23]. However, evidence also exists suggesting that application of ColVI may have an overall fibrotic effect alongside its mitogenic potential [26,27].

Despite these findings in other biologic systems, to our knowledge no studies have been performed to investigate the role of soluble ColVI in IVD degeneration. Thus, the aim of the current study was to further characterize the role of ColVI in IVD degeneration and to examine the effect of soluble ColVI on degenerative NP cells.

## 2. Materials and Methods

### 2.1. Human Samples

Forty-seven human IVD tissue samples were obtained from patients undergoing anterior lumbar interbody fusion, under a protocol approved by the Institutional Review Board at our institution (08D.525). One sample was harvested post-mortem. Samples harvested intraoperatively were placed into specimen cups with 0.9% saline and transferred on ice to the laboratory within 30 min of harvest to minimize degradation. Normal NP tissue was obtained from post-mortem samples within 24 h of donor death from the Cooperative Human Tissue Network (University of Pennsylvania, Philadelphia, PA, USA). Each specimen came from one donor. Disc tissue was immediately separated into NP and AF by gross examination. Specimens were rinsed with phosphate-buffered saline (PBS) and then washed once again in PBS, 1 mM phenylmethanesulfonyl fluoride (PMSF), and a protease inhibitor cocktail (Complete Mini; Roche Diagnostics, Indianapolis, IN, USA). For RNA isolation, tissues were stabilized in RNA*later* solution (Ambion, Life Technologies, Grand Island, NY, USA) following the manufacturer’s instructions and stored at −80 °C. IVDD was graded on sagittal T2 magnetic resonance imaging (MRI) images via Pfirrmann classification (Supplementary Table S1) [28].

### 2.2. Isolation of NP Cells

Human NP cells were isolated using a method described previously by our lab [29]. NP cells were digested using an enzyme solution containing 0.4% Pronase (Calbiochem Roche Diagnostics, Indianapolis, IN, USA) for 20 min with gentle stirring in a 37 °C, 5% CO_2_ tissue culture incubator and then incubated with 0.025% Collagenase P (Roche Diagnostics, Indianapolis, IN, USA) for 16 h. At the end of the digestion period, the tissue was washed 3 times with sterile medium. Isolated NP cells were cultured in complete culture medium supplemented with 10% fetal bovine serum (FBS). Only P0 and P1 passages of cells were used for analysis.

### 2.3. Treatment

NP cells were treated with 5 or 10 μg/mL soluble ColVI (Corning Inc., Corning, NY, USA) for 48 h. For the inhibitor assay, cells were pretreated for 1 h with the MEK/ERK inhibitor PD98059 (25 μM) (Merck KGaA, Darmstadt, Germany) followed by incubation with ColVI. Preliminary experiments indicated that these concentrations elicited measurable cellular responses without affecting cell viability. These concentrations lie within the range that has been reported to induce the biologic effects of ColVI in other cell types [23,30,31]. All the experiments were independently carried out 3 times.

### 2.4. RNA Extraction and Quantitative Real-Time PCR

Total RNA from tissue and from NP cells was isolated using methods described previously [29,32]. Purified, DNA-free RNA was converted to complementary DNA (cDNA) using RNA to cDNA EcoDry Premix (Oligo dT, Clontech Laboratories Inc., Mountain View, CA, USA) and quantitative real-time polymerase chain reaction (PCR) was performed using SYBR Green PCR Master Mix (Applied Biosystems Life Technologies, Grand Island, NY, USA) on a StepOne Real-Time PCR System. Melting curve analysis was performed to ensure a specific PCR product while excluding primer dimers. The amount of PCR product was estimated using a relative standard curve quantification method. The data were normalized to the expression level of glyceraldehyde-3-phosphate dehydrogenase (GAPDH) or to the expression level of hypoxanthine phosphoribosyl transferase 1 (Hprt1). All the primers were synthesized by Integrated DNA Technologies (Coralville, IA, USA).

### 2.5. Cell Proliferation Assay (WST-1)

To elucidate ColVI-induced signaling events, human NP cells (*n* = 5) were exposed to soluble ColVI. Proliferation assays were performed using the WST-1 kit (Takara Bio Inc., Shiga, Japan). Cells were plated in 96-well plates at 1 × 10^4^ cells per well in 100 μL DMEM with 10% FBS culture medium. After 24 h, cells were washed twice with serum-free Opti-MEM + Gluta Max-I media (Invitrogen/GIBCO) (starving medium) and then cultured for 24 h to synchronize cell cycles. After 24 h, the medium was changed to 1.0% FBS containing 5 or 10 μg/mL human soluble ColVI (Corning Inc., Corning, NY, USA) and the cells were treated for 48 h. Premixed cell proliferation reagent was added to each well (10 μL/well) and the plates were incubated at 37 °C for 1 h before measuring the absorbance at 450 nm using a Synergy HT microplate reader (BioTek Instruments, Inc., Winooski, VT, USA). These experiments were repeated a minimum of three times.

### 2.6. Protein Extraction and Western Blot

After treatment, NP cells were placed immediately on ice and washed with ice-cold PBS. Total protein was extracted with lysis buffer (Cell Signaling Technologies, Danvers, MA, USA) and a protease inhibitor cocktail (Complete Mini; Roche Diagnostics, Indianapolis, IN, USA) at 4 °C for 30 min. The lysate was centrifuged for 10 min at 14,000× *g* to collect the clear extract. Protein concentration was determined using a Pierce BCA protein assay kit (Thermo Fisher Scientific Inc., Rockford, IL, USA). Equal amounts of protein (10 µg) were electrophoresed on NuPAGE 4–12% Bis-Tris Gels (NOVEX by Life Technologies, Carlsbad, CA, USA) and then transferred electrophoretically to Immobilon-P Membrane (Millipore, Billerica, MA, USA). After blocking with 5% nonfat dry milk in PBS with 0.1% Tween-20 at room temperature for 1 h, membranes were incubated overnight at 4 °C in 5% BSA in PBS with 0.1% Tween-20 with primary antibodies at 1:1000 dilutions on a rocking platform. Anti-p44/42 MARK (ERK1/2), anti-phosphorylated p44/42 MARK (ERK1/2) (Thr202/Tyr204), anti-Akt (pan) (C67E7), anti-phosphorylated Akt (Ser473), anti-Stat3 (124H6) and anti-phosphorylated Stat3 (Tyr705) (D3A7) were utilized (Cell Signaling Technology, Boston, MA, USA). The blots were incubated with horseradish-labeled secondary antibodies (anti-rabbit IgG or anti-mouse IgG) at 1:10,000 dilutions for 1 h at room temperature and binding of the secondary antibody was detected by enhanced chemiluminescence (ECL-Plus, GE Healthcare, Life Technologies, Carlsbad, CA, USA). After each antibody, the membrane was stripped (Restore™ Western Blot Stripping Buffer, ThermoFisher Scientific, Rockford, IL, USA) and reprobed with the next one.

### 2.7. Microarray Analysis

After treatment with 10 μg/mL soluble ColVI for 48 h, the NP cells were collected, RNA was isolated and gene expression analysis was performed using GeneChip^®^ Human Transcriptome Array 2.0 (Affymetrix, Santa Clara, CA, USA) chip arrays to identify differential gene expression between cells treated with ColVI and untreated control (CTR).

### 2.8. Immunocytochemistry

NP cells were cultured on chambered slides in DMEM with 10% FBS for 24 h. After being washed, cells were starved for 24 h in Opti-MEM + Gluta Max-I medium and were incubated with or without 10 μg/mL soluble ColVI for 10 min. After the incubation, cells were fixed with ice-cold methanol for 20 min at −20 °C and blocked with 10% Goat serum, 1× TBS, and 0.004% Tween-20 for 1 h. Fixed cells were incubated with anti-phosphorylated p44/42 MAPK (ERK1/2) (1:50) and anti-phosphorylated STAT3 (1:100) antibodies diluted in 10% Goat serum/PBS overnight at 4 °C. Bound antibodies were detected using goat anti-rabbit IgG Antibody, Cy3 conjugate (1:1000, EMD Millipore, Billerica, MA, USA). Digital images were acquired using a Nikon Ti Eclipse confocal microscope (Carl Zeiss Microscopy GmH, Göttingen, Germany).

### 2.9. Data Analysis

Statistical analysis was performed using Sigma Plot 11.2 statistical software (Systat Software, Inc., San Jose, CA, USA). Student’s *t*-test was applied to determine the difference between the untreated group and individual treatment groups. Significance was determined to be a *p*-value < 0.05.

## 3. Results

### 3.1. RT-PCR Analysis of Isolated NP Cells

**Figure 1:**
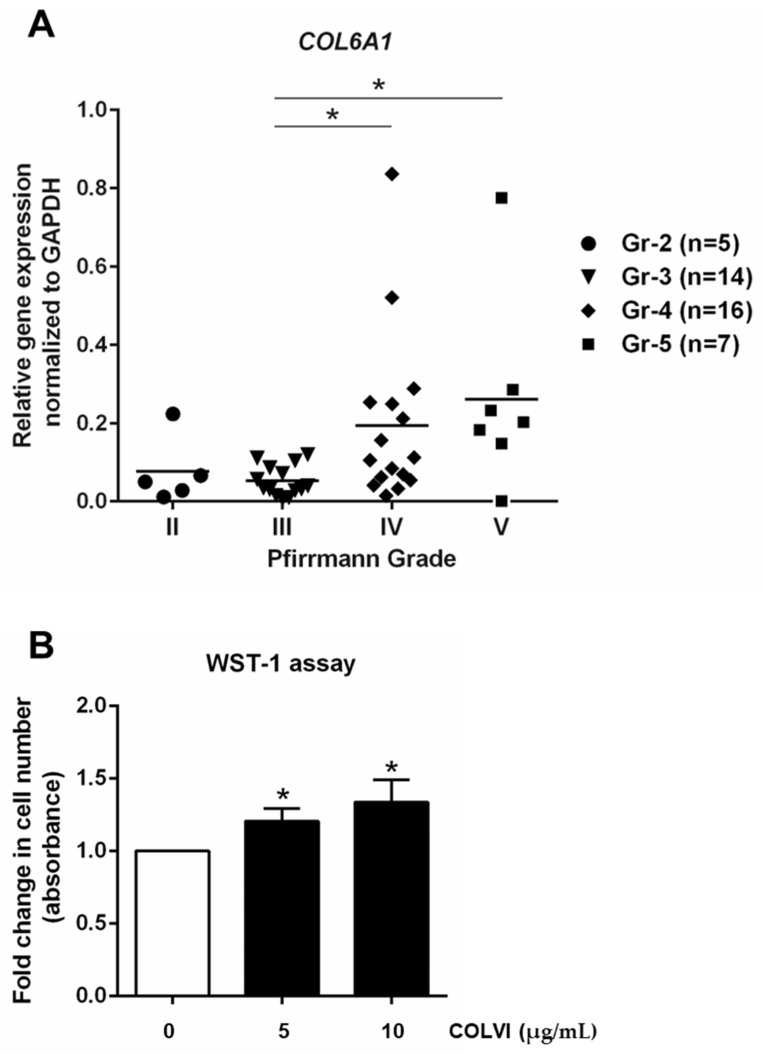
*COL6A*1 is highly expressed in human degenerated IVDs. (**A**) Quantitative RT-PCR. The level of mRNA expression of *COL6A1* is significantly elevated in the NP tissues with disc degeneration compared to the Gr-3 group. In the legend, “*n*” represents the number of biological replicates. Data represent the mean in expression normalized to GAPDH (±SD). (**B**) Effect of soluble ColVI on the proliferative ability of untreated control NP cells (*n* = 5). The cells were cultured for 48 h in the presence of ColVI at various concentrations and were repeated in triplicate. Here, the data is represented as a mean fold change (±SD). A Student’s *t*-test was utilized to determine significance (* denotes a *p*-value < 0.05).

Compared to Pfirrmann grade III, COL6A1 mRNA increased 4.4-fold (*p* = 0.024) in grade IV, and 5.3-fold (*p* = 0.014) in grade V cells (Figure 1A).

### 3.2. Cell Proliferation Assay of NP Cells Treated with Soluble ColVI

Cell proliferation increased 1.204-fold (*p* < 0.001) in NP cells treated with 5 µg/mL ColVI and 1.335-fold (*p* = 0.001) in those treated with 10 µg/mL ColVI compared to untreated cells (Figure 1B).

### 3.3. Gene Regulation in NP Cells Treated with ColVI

**Figure 2:**
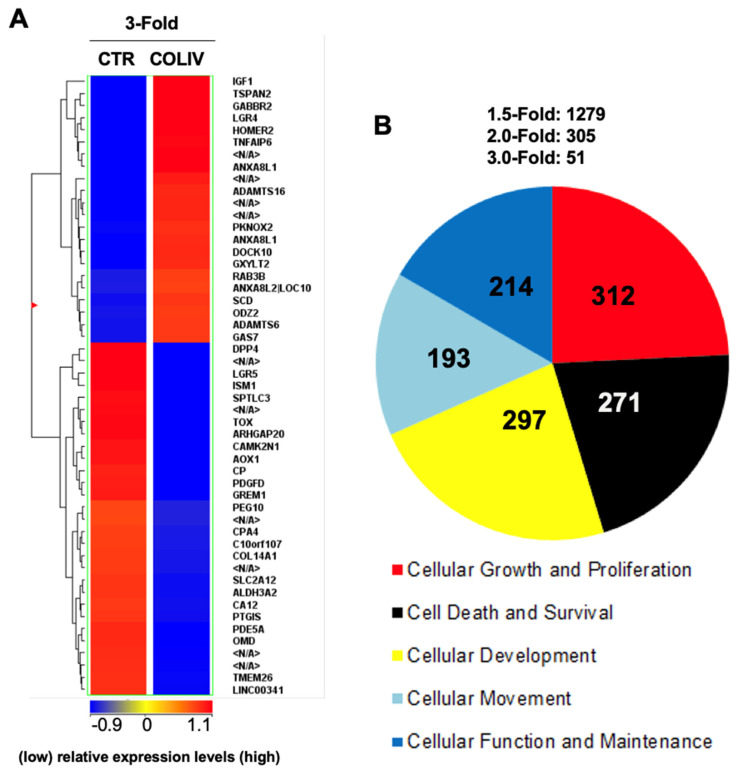
Microarray analysis of NP cells treated with soluble ColVI. (**A**) The heat map displays gene expression patterns of 51 genes (3-fold) after treatment. The relative levels of gene expression are depicted with a color scale, where red represents the highest level of expression and blue the lowest. (**B**) Pie chart showing differentially expressed genes based on major molecular and cellular functions. The number represents genes associated with a specific function (1.5-Fold: 1279 genes). Data shown is from 1 experiment.

1279 genes were upregulated or downregulated by more than 1.5-fold in ColVI-treated cells. *IGF1* (7.9-fold), *TSPAN2* (5.3), *GABBR2* (5.2), *HOMER2* (4.9), and *LGR4* (4.9) were upregulated, whereas *LGR5* (−6.7-fold), *ISM1* (−6.6), *DPP4* (−5.6), *TOX* (−4.5), and *ARHGAP20* (−4.5) were downregulated (Table 1, Figure 2A).

**Figure 3:**
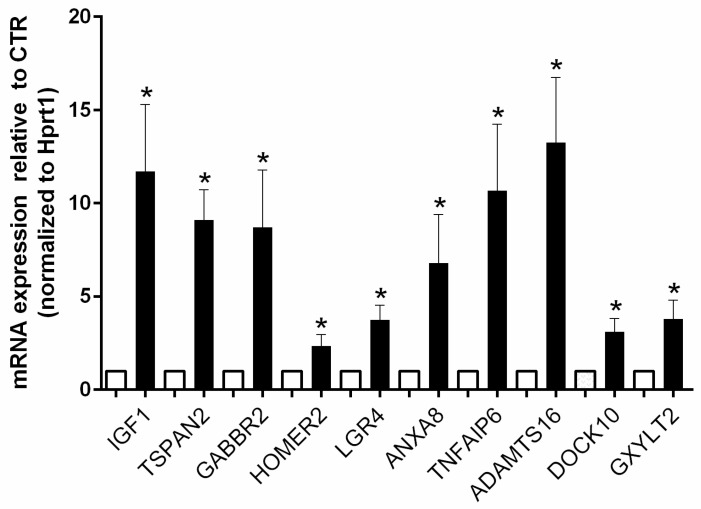
Results of Quantitative RT-PCR validation of Microarray analysis of NP cells treated with 5 µg/mL soluble ColVI. Data is represented as the mean mRNA expression relative to the untreated controls (±SD). Three independent experiments were used for this analysis. Student’s *t*-test was utilized to compare the relative expression of a particular protein from an untreated sample to a treated one, with a * denoting significance (*p*-value < 0.05).

qRT-PCR validation was performed on selected genes identified in the microarray analysis utilizing three independent biologic replicates (Figure 3). When treated with 10 µg/mL ColVI, the upregulated genes had significantly increased mRNA expression compared to untreated (Figure 3).

### 3.4. Activation of ERK, Akt, and Stat3 Pathways

**Figure 4:**
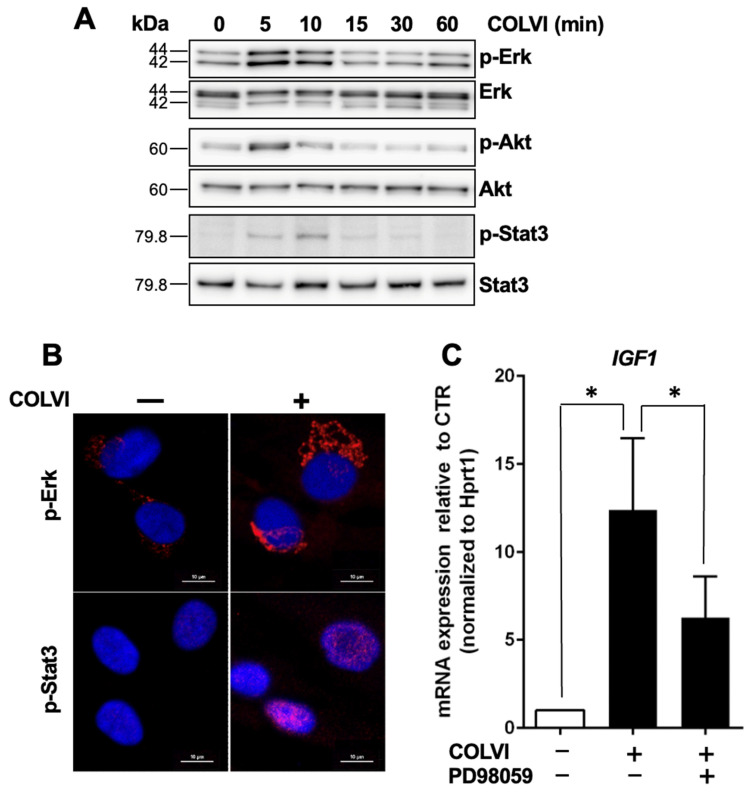
(**A**) Western blot analysis. Total cellular extracts were analyzed for phosphorylation and expression of ERK, Akt and Stat3 (*n* = 3). Phosphorylation peaks at around 5–10 min after treatment with ColVI. This gel represents one experiment. These results should be interpreted qualitatively as they were not quantitatively analyzed. (**B**) Immunocytochemistry. Serum-starved NP cells on chambered slides were treated with 5 µg/mL ColVI for 30 min. After fixation, cells were stained with polyclonal anti-Phospho-ERK and anti-Phospho–Stat3 and secondary anti-rabbit IgG Cy3 conjugate antibody. Addition of ColVI induced phosphorylation of ERK and Stat3 and translocation into the nucleus. Scale bars, 10 μm (120×) (**C**) Quantitative RT-PCR analysis (*n* = 3). The treatment of PD98059 significantly decreased mRNA levels of *IGF1* and neutralized the effect of ColVI in NP cells. Data represent the mean mRNA expression relative to untreated NP cells ± SD. Each independent experiment was repeated three times. Student’s *t*-test determined significance (denoted by a * for a *p*-value < 0.05).

Representative western blot suggests that phosphorylation of ERK, Akt, and Stat3 increased after exposure to ColVI (Figure 4A). Addition of ColVI induced phosphorylation of ERK and Stat3 and nuclear translocation (Figure 4B). ColVI treatment of NP cells showed a significant increase in *IGF-1* mRNA (12.4, *p* < 0.001) expression compared to untreated NP cells. Furthermore, co-treatment of NP cells with ColVI and ERK inhibitor (PD98059) showed a 6.2-fold increase in *IGF-1* mRNA, which was significantly lower than that produced by NP cells treated with ColVI alone (*p* = 0.009) (Figure 4C).

## 4. Discussion

Research has increasingly implicated changes in gene expression and polymorphisms in the pathogenesis of IVDD [5]. A potential therapeutic target in cartilage systems outside of the spine is ColVI, which is uniquely enriched in the PCM surrounding chondrocytes [7,11,12]. Additionally, soluble ColVI has been shown to enhance mesenchymal stem cell expansion and chondrocyte proliferation in non-spine studies [23,30,31]. The current work is the first to investigate the impact of soluble ColVI on NP cells.

Increased Pfirrmann grade was associated with *COL6A1* upregulation by NP cells. The addition of soluble ColVI to NP cells in vitro was associated with increased proliferation and marked upregulation of IGF-1. Soluble ColVI also led to increased phosphorylation of ERK, Akt, and Stat3. Importantly, co-treatment of NP cells with both ColVI and ERK inhibitor PD98059 significantly decreased IGF-1 mRNA production compared to treatment with ColVI alone.

The finding of increased *COL6A1* expression in IVDs with higher degeneration grade is consistent with prior work. Watanabe et al. noted that rabbit disks in the early stages of degeneration showed greater ColVI staining compared to collagen Type II [33]. Additionally, when degeneration was delayed via co-culture with NP cells, ColVI appeared later—the authors concluded that ColVI expression was actively associated with degeneration [33]. Nerlich et al. noted that in several anatomic regions of human lumbar disks, there was decreased Type II collagen and increased ColVI with increased age, structural disorganization, and degeneration [34]. Finally, the *COL6A2* gene has been identified as a hub gene for IVD degeneration, meaning that it acts as a central node for other genes in this process [17]. The extent to which ColVI acts as a marker versus as a driver of the pathogenesis of IVD degeneration requires further research to clarify [17,18,19,20,21,22].

ColVI increased NP cell proliferation. While IVDD is characterized by a net decrease in NP chondrocyte stock, cell proliferation and clustering as a tissue regenerative response has also been noted [35]. Thus, our observation that soluble ColVI improves NP proliferation may be viewed as the product of ColVI promoting the natural history of IVDD [36]. This latter interpretation could be supported by our finding that suggests soluble ColVI upregulates IGF-1. This growth factor and its receptor are overexpressed during NP proliferation associated with IVDD [36]. Ostensibly, increased growth factor signaling should be associated with tissue repair and NP population expansion [37]. However, prior work has suggested that within the nutrient-depleted environment of IVDD, increased growth factor signaling further increases nutrient demand and thus worsens the energy and anabolic/catabolic balance within the disc [37]. IGF-1 especially has been noted to cause “misguided” cellular proliferation, which increases nutritional demand and consequent disc deterioration [38]. Additionally, NP proliferation in response to degenerative growth factor signaling leads to cell clustering rather than reconstitution of the native disc chondrocyte formation [35].

Our preliminary results suggest that soluble ColVI may lead to ERK, Akt, and Stat3 phosphorylation, which could support ColVI’s role in growth factor signaling associated with IVDD. Additionally, the impact of soluble ColVI on IGF-1 expression was substantially attenuated by the addition of a specific ERK inhibitor, which may support the relationship between ColVI and ERK. ERK and Akt pathways have been noted in prior work as critical to growth factor transduction of mitogenic signaling in IVD cells [39,40,41]. Additionally, it has been postulated that *COL6A2* may drive IVDD by regulating Akt signaling, which has downstream effects on intracellular glucose metabolism [22]. Specifically, increased Akt signaling can reduce glucose storage and increase glycolysis to promote cellular proliferation [17]. Thus, it appears that soluble ColVI acts on degenerative NP cells to increase IGF-1 growth factor signaling, likely via ERK and potentially through Akt and Stat3 pathways, to promote NP proliferation. Given that prior literature suggests that growth factor signaling such as this in the setting of IVDD further accelerates disc deterioration, it is unlikely that soluble ColVI itself represents a promising therapy. Instead, our work may suggest that ColVI is at least a biomarker of disc degeneration. Elucidating the relationship of the IGF-1/ERK pathway and ColVI-induced proliferation, and whether inhibition of its mediators leads to delayed or absent IVDD, could provide helpful therapeutic targets.

The current work has several limitations. First, we did not record the ambulatory status, BMI, smoking status, diabetes, medication use, or genetic profiles of the patients or cadavers prior to surgery or sample acquisition. These factors may have had occult effects on the disc cells that were not able to be evaluated. The observed transcriptional differences between treated and untreated NP cells could be, in part, due to genetic variation between donors. A greater number of included patients is needed to perform more granular subgroup analysis and to homogenize the study population. With regards to our available data, we only have one representative microarray, which can only describe a trend in the genetic profile, and we also only have qualitative results for our western blot. We were also not able to fully evaluate the downstream implications of each gene expression change noted—future work may be aimed at targeting and further characterizing the effects of these other genes impacted by soluble ColVI. We focused on IGF-1 because it was the most robustly elevated and because it had been previously described within the context of ERK and Akt signaling. Similarly, we only tested inhibition of one pathway (ERK) on IGF-1 expression in ColVI-exposed NP cells. Measuring the change in NP proliferation after inhibiting ERK or other pathways (Akt or Stat) could help provide more robust information on the ColVI-induced proliferation mechanism.

## 5. Conclusions

The current work is among a small body of literature exploring the role of ColVI in IVDD and so far, the only work evaluating the impact of soluble ColVI on NP cells. A greater degree of disc degeneration was associated with increased *COL6A1* expression. Soluble ColVI increased NP proliferation and greatly upregulated IGF-1. Preliminary results suggest that exposure to ColVI increased the phosphorylation of ErK, Akt, and Stat3. The effect of soluble ColVI on IGF-1 was significantly attenuated by the addition of an ERK inhibitor, suggesting that this pathway is mediated by ERK signaling. Within the context of prior literature, these findings suggest that ColVI may play a role in attempted NP regeneration and tissue repair via growth signaling. However, it is likely that these effects drive further energy imbalance, cell clustering, and ultimately deterioration of the degenerative phenotype. The next steps should include clarifying the role of the IGF-1/ERK on ColVI-induced NP proliferation as well as targeting downstream mediators, which may slow or prevent IVDD.

## Figures and Tables

**Table 1:** Top ten genes that demonstrated the highest upregulation and downregulation in NP cells treated with soluble Col VI.

Symbol	Gene Description	Expression Levels
*IGF1*	Insulin-like growth factor 1(somatomedin C)	7.895
*TSPAN2*	Tetraspanin2	5.281
*GABBR2*	Gamma-aminobutyricacid (GABA)B receptor,2	5.160
*HOMER2*	Homerhomolog2 (Drosophila)	4.914
*LGR4*	Leucine-rich repeat-containing G protein-coupled receptor4	4.871
*ANXA8/ANXA8L1*	AnnexinA8-like1	4.712
*TNFAIP6*	Tumor necrosis factor, alpha-induced protein6	4.524
*ADAMTS16*	ADAM metallopeptidase with thrombospondin type1 motif,16	3.660
*DOCK10*	Dedicator of cytokinesis10	3.624
*GXYLT2*	Glucosidexylosyl transferase2	3.594
*LGR5*	Leucine-rich repeat-containing G protein-coupled receptor 5	−6.950
*ISM1*	Isthmin1 homolog (zebrafish)	−6.615
*DPP4*	Dipeptidyl-peptidase4	−5.643
*TOX*	Thymocyte selection-associated high mobility group box	−4.486
*ARHGAP20*	Rho GTPase activating protein20	−4.467
*SPTLC3*	Serine palmitoyltransferase, long-chain base subunit3	−4.287
*AOX1*	Aldehydeoxidase1	−4.111
*CAMK2N1*	Calcium/calmodulin-dependent protein kinase II inhibitor1	−4.091
*GREM1*	gremlin1	−3.890
*PDGFD*	Platelet-derived growth factor D	−3.870

## Data Availability

The raw data supporting the conclusions of this article will be made available by the authors on request.

## Supplementary Materials

The following supporting information can be downloaded at https://www.mdpi.com/article/10.3390/cells15151339/s1: Supplementary Table S1. Details for human tissues used in Real-time PCR and Immunohistochemical analysis. PM: Postmortem samples; M: Male; F: Female; L: Lumbar.

## Abbreviations

The following abbreviations are used in this manuscript:

ColVI	Collagen VI
ECM	Extracellular matrix
IVDD	Intervertebral disc degeneration
IVD	Intervertebral disc
DDD	Degenerative disc disease
NP	Nucleus Pulposus
AF	Annulus Fibrosus
LBP	Lower back pain
