# Supplementary material for: The Biological Response of Human NP Cells to Soluble Collagen VI
Source: Cells. 2026 Jul 26;15(15):1339. doi: 10.3390/cells15151339 (PMC13465566; doi:10.3390/cells15151339)
Supplement: Supplementary file 1 [file cells-15-01339-s001.zip › cells-4336859-supplementary.pdf]

Supplemental Table S1: Details for human tissues used in Real-time PCR and Immunohistochemical analysis. PM: Postmortem samples; M: Male; F: Female; L: Lumbar.

	Pathology	Sex	Age (yr)	Disc level	Pfirrmann Grade	RT-PCR	Cell Isolation
1	Spondylolisthesis	F	49	L4-L5	2	X	
2	Scoliosis	F	40	L3-L4	2	X	
3	DDD	M	33	L5-S1	2	X	
4	DDD	F	48	L5-S1	2	X	
5	DDD	M	43	L2-L3	2	X	
6	Trauma	M	26	T12-L1	3	X	
7	Trauma	M	51	L1-L2	3	X	
8	Scoliosis	M	26	L2-L3	3	X	
9	DDD	F	61	L4-L5	3	X	
10	DDD	M	38	L4-L5	3	X	
11	DDD	M	45	L5-S1	3	X	
12	Scoliosis	F	40	L4-L5	3	X	
13	DDD	M	41	L4-L5	3	X	
14	Scoliosis	F	40	L2-L3	3	X	
15	DDD	M	54	L4-L5	3	X	
16	DDD	M	38	L4-L5	3	X	
17	DDD	F	30	L5-S1	3	X	
18	DDD	M	44	L4-L5	3	X	
19	DDD	M	39	L4-L5	3	X	
20	DDD	M	45	L4-L5	4	X	
21	DDD	F	46	L4-L5	4	X	

22	DDD	M	44	L5-S1	4	**X**	
23	DDD	M	38	L5-S1	4	**X**	
24	DDD	M	40	L5-S1	4	**X**	
25	DDD	M	41	L3-L4	4	**X**	
26	DDD	F	24	L4-L5	4	**X**	
27	DDD	F	47	L4-L5	4	**X**	
28	DDD	M	58	L5-S1	4	**X**	
29	DDD	M	38	L5-S1	4	**X**	
30	Trauma	M	65	L2-L3	4	**X**	
31	DDD	F	64	L3-L4	4	**X**	
32	DDD	F	64	L4-L5	4	**X**	
33	DDD	F	31	L4-L5	4	**X**	
34	DDD	F	30	L4-L5	4	**X**	
35	DDD	F	43	L4-L5	4	**X**	
36	DDD	M	64	L4-L5	5	**X**	
37	DDD	F	43	L4-L5	5	**X**	
38	DDD	F	64	L5-S1	5	**X**	
39	DDD	F	57	L5-S1	5	**X**	
40	DDD	F	54	L5-S1	5	**X**	
41	DDD	F	43	L5-S1	5	**X**	
42	DDD	F	31	L5-S1	5	**X**	
43	DDD	F	40	L5-S1	4		**X**
44	DDD	F	63	L5-S1	3		**X**
45	DDD	F	60	L4-L5	3		**X**

46	DDD	F	28	L4-L5	4		**X**
47	DDD	M	55	L3-L4	5		**X**
48	PM	F	33	L4-L5	None (autopsy)		**X**
